# Modulatory effects of trophoblast-secreted CXCL12 on the migration and invasion of human first-trimester decidual epithelial cells are mediated by CXCR4 rather than CXCR7

**DOI:** 10.1186/s12958-018-0333-2

**Published:** 2018-03-02

**Authors:** Jiayi Zheng, Haiping Wang, Wenhui Zhou

**Affiliations:** Medical Center for Human Reproduction, Beijing Chaoyang Hospital, Capital Medical University, Beijing, 100020 People’s Republic of China

**Keywords:** CXCL12/CXCR4/CXCR7, Decidual epithelial cell (DEC), Trophoblast (TC), Maternal-fetal crosstalk

## Abstract

**Background:**

Maternal-fetal crosstalk during embryo implantation is complex and regulated by local signaling molecules. Chemokines and their receptors are critical signaling components required for implantation and the process of pregnancy. This study aimed to explore whether human first-trimester trophoblast cells (TCs) were capable of modulating the migration and invasion of human first-trimester decidual epithelial cells (DECs) via CXCL12/CXCR4/CXCR7 signaling.

**Method:**

The expression of CXCR4 and CXCR7 in DECs was examined by immunohistochemistry, immunocytochemistry, immunofluorescence, flow cytometry, real-time polymerase chain reactions and western blotting. The effects of recombinant human CXCL12 (rhCXCL12) and TC-conditioned medium (TC-CM) on DEC viability in vitro were explored using a viability assay. The modulatory effects of rhCXCL12 and TC/DEC co-cultures on DEC migration and invasion were examined with migration/invasion assays.

**Result:**

CXCR4 and CXCR7 were co-expressed in human first-trimester DECs. Human rhCXCL12 and TC-CM had no effects on DEC viability in vitro (*P* > 0.05). Both exogenous CXCL12 and co-culture with TCs significantly increased the migration and invasion of DECs (*P* < 0.05). Neutralizing antibodies against CXCR4 (P < 0.05) or CXCL12 (P < 0.05), but not CXCR7 (*P* > 0.05), significantly blocked the enhanced migration and invasion of DECs induced by exogenous CXCL12 or TC co-culture.

**Conclusions:**

CXCR4 and CXCR7 were co-expressed in human first-trimester DECs. TC-derived CXCL12 promoted the migration and invasion of DECs via CXCR4, but not CXCR7, in a paracrine manner during early pregnancy.

## Background

The application of assisted reproductive technology (ART) in the clinic has made great achievements in infertility treatment. However, even when embryos/blastocysts estimated to be top grade are transferred, the occurrence of pregnancy failure is still inevitable, suggesting the importance of implantation [1, 2].

Successful implantation requires the perfect coordination between the fetal-derived trophoblasts (TCs) and maternal-derived decidual cells via a complex network of molecules signaling in an autocrine and paracrine manner. Decidual epithelial cells (DECs), serving as a critical modulator for conceptus attachment, have been ignored for many years. In addition to providing nutrition and energy for the fetus, decidual epithelial cells have gradually been shown to be involved in immune regulation, TC invasion, protection against infections, and the production of growth factors and cytokines [3–8]. Deficient glandular activity has been observed in some recurrent miscarriages, further implying an important role for endometrial glands in pregnancy [6].

DECs have gradually attracted increasing attention in recent years. However, due to the difficulty in isolating and culturing primary cells, there is limited information available about the interaction between TCs and decidual glands. The motility of DECs during implantation and early pregnancy is still an open question.

Chemokine (C-X-C motif) ligand 12 (CXCL12) is a small cytokine. CXCL12 and its receptors CXCR4/CXCR7 are involved in several crucial reproductive biology processes, including uterine natural killer cell recruitment, placentation, implantation and embryogenesis [9–12].

Our team focuses on the roles of chemokines and chemokine receptors in the maternal-fetal dialogue. We have confirmed that human first-trimester TCs not only modulate their own invasiveness via the CXCL12/CXCR4 axis but also promote the motility of DSCs via CXCR4 ligation [12, 13]. Our recent research further revealed the expression of CXCR7 (another high affinity receptor for CXCL12) in human first-trimester TCs and DSCs [14, 15]. Moreover, steroid hormones showed priming effects on CXCR4 and CXCR7 expression in human cycling and early pregnant DSCs before the emergence of an embryo [14, 15]. These results indicate the potential importance of the CXCL12/CXCR4/CXCR7 axis in implantation and early pregnancy. With the discovery of CXCR7, researchers may need to re-examine the role of the CXCL12/CXCR4 signaling pathway. Our knowledge of the expression and function of CXCR7 at the maternal-fetal interface and its relation to CXCL12/CXCR4 signaling remains to be elucidated.

The aims of this study are first, to examine CXCR4 and CXCR7 expression in human first-trimester DECs, and second, to determine the effects of TC-derived CXCL12 on DEC functions. We hope this study improves our understanding of the detailed mechanisms underlying the maternal-fetal dialogue during implantation and early pregnancy and provides new clues for the diagnosis and treatment of implantation failure-related infertility and early miscarriage.

## Methods

### Tissue collection

As previously described, [12, 13, 15] human decidual and villous tissues were collected from patients who underwent an elective surgical abortion in first-trimester pregnancies (gestational age 6–10 weeks) for non-medical reasons at Beijing Chaoyang Hospital from 2015 to 2016. Samples were collected in ice-cold DMEM (Dulbecco’s Modified Eagle’s Medium; Gibco), then immediately transported to the laboratory and separated for paraffin embedding or cell isolation [12, 13, 15]. This study was approved by the Human Research Ethics Committee of Beijing Chaoyang Hospital. All participants completed an informed consent form prior to the collection of tissue samples.

### Immunohistochemistry

The method for IHC was previously established in the laboratory [12, 16, 17]. Briefly, the decidual tissues were routinely fixed with 4% formalin and embedded in paraffin. Antigen retrieval was performed by microwave heating in 0.01 M sodium citrate buffer (pH 6.0) at 800 W for 3 min to initiate boiling and then for 7 min at 400 W to continue boiling. After cooling down to the room temperature, the 10 min heating procedure was repeated with fresh buffer. H_2_O_2_ (0.3%) in phosphate-buffered saline (PBS) was employed to block endogenous peroxidase activity in the sections. After treatment with a protein blocking solution containing 5% bovine serum albumin to block non-specific binding, sections were incubated with mouse anti-human CXCR4 (25 μg/ml), CXCR7 (20 μg/ml) monoclonal antibody (Catalogue number MAB172/MAB4227, R&D Systems, Abingdon, UK) or mouse immunoglobulin (Ig)G isotype control (20 μg/ml, Sino-America Co. Ltd., Shanghai, China) overnight at 4 °C. A streptavidin/biotin detection reagent kit (ZSGB-BIO Co. Ltd., Beijing, China) with 3, 30-diaminobenzidine tetrahydrochloride (DAB) was employed to detect signals, and Harris hematoxylin was used as counter-stain. The expression of CXCL12, CXCR4 and CXCR7 in human first-trimester TCs has been confirmed in our previous study [12–14]. According to an ELISA, the concentration of accumulated CXCL12 in TC-CM increased constantly in vitro [12]. In the present study, human first-trimester TCs were used as positive control. Mouse isotype IgG was used as negative controls. The experiments were perform using ten independent samples.

### Isolation and culture of human first-trimester DECs

DECs were isolated according to the method established in our laboratory [18]. Endometrial tissues were digested independently with 0.1% collagenase IV for 1 h at 37 °C on the shaker. The resulting digest was passed through a 38 μm sieve. Then, the reverse side of the 38 μm sieve was washed with DMEM, which was collected and centrifuged at 1000 g for 15 min. The pellet was diluted with DMEM containing 15% heat-inactivated fetal bovine serum (FBS) and seeded in a culture flask. After 4 h of culture, the suspension containing non-attached cells was collected and seeded on 6-well plates pre-coated with extracellular matrix (ECM; Sigma, St. Louis, MO, USA). With this method, the purity of the cultured DECs can reach to 90% according to ICC identification (positive for CK7 and negative for vimentin) [18].

### Isolation and culture of human first-trimester TCs

Villus tissue was treated by repeated trypsin digestions according to the method previously established [12–14]. Briefly, placental tissues were collected and digested with 0.25% trypsin (Amresco) and 2.5 K units/ml DNase type I (Sigma, St. Louis, MO, USA) for 10 min at 37 °C with gentle agitation. The digested cell suspension was collected and the residual tissue was subjected to two to three additional cycles of 10 min digestions. Cell suspensions obtained from each digestion were mixed, carefully layered over a discontinuous Percoll gradient (65–20%, in 5% step), and thereafter centrifuged at 1000 g for 20 min. Cells sedimenting at densities between 1.048 and 1.062 g/ml were collected and washed with DMEM. These cells were then diluted at a density of 5 × 10^5^ cells/well, and maintained in complete DMEM (2 mM glutamine, 25 mM HEPES, 100 IU/ml penicillin and 100 mg/ml streptomycin), supplemented with 15% FBS and incubated in a 5% CO_2_ incubator at 37 °C. This method yields 95% pure TCs, the characterization of TCs is reported in a previous publication [12].

### Immunocytochemistry

The method for ICC was previously established in the laboratory [12, 14]. Briefly, the isolated DECs and TCs were cultured for 24–48 h, before being fixed with 4% paraformaldehyde and washed with PBS. After treatment with 0.3% H_2_O_2_ in PBS to block endogenous peroxidase activity, cells were blocked with 7% horse serum in PBS and incubated with mouse anti-human CXCR4 and CXCR7 monoclonal antibodies. The antibodies used, as well as the other procedures, were identical to the described above for IHC. Experiments were performed using five independent TC and DEC preparations in each.

### Immunofluorescence

Immunofluorescence was used to detect the expression and position of CXCR7 and CXCR4 in DECs. The isolated DECs were seeded on 6-well plates which were pre- coated with cover glasses at a density of 2 × 10^6^ cells/ml × 2 ml per well. At 80–90% confluence, the cells were fixed in 4% paraformaldehyde and treated with 0.2% Triton X-100. After blocking with goat serum for 2 h at room temperature, the cells were incubated with mouse anti-human CXCR4 (25 μg/ml, catalogue number MAB172, R&D Systems, Abingdon, UK) and rabbit anti-human CXCR7 monoclonal antibodies (20 μg/ml, catalogue number ab72100, Abcam, Cambridge, MA,). After three washes with PBS, the cells were incubated with fluoresce-in isothiocyanate (FITC) conjugated donkey anti-rabbit immunoglobulin (Ig) G (1:100, 10 μg/L) or Texas Red conjugated donkey anti-mouse IgG (1:100, 10 μg/L) (Rockland) at room temperature for 60 min in darkness and then incubated with DAPI (Santa Cruz Biotechnology) for 5 min at 37 °C. Images were captured with an Olympus fluorescence microscope (Olympus, Tokyo, Japan). The experiments were perform using three samples.

### Enzyme-linked immunosorbent assay

As previously described, [12] purified TCs were seeded in a 24-well plate pre-coated with matrigel at a density of 5 × 10^5^ cells/ml. Trophoblast supernatants were collected after 24, 48 and 72 h of culture. Each supernatant was centrifuged at 2000 g and stored at − 70 °C. A human CXCL12 Quantikine enzyme-linked immunosorbent assay (ELISA) kit (Quantikine, R&D Systems, DSA00) was used to measure chemokine levels in each supernatant, according to the manufacturer’s instructions. The intra-assay coefficient of variation is 3.5%, inter-assay coefficient of variation is 9.6%. The average value of each sample was used for statistical analysis. The ELISA was conducted in duplicate in three separate experiments.

### Real-time PCR

Total cellular RNA was extracted from primary DEC cultures and reverse transcribed, according to our previously described method [12, 15]. Brifly, Total RNA was extracted from cells by Trizol (Invitrogen). The reverse transcription reaction was performed using Fast Quant RT Kit (with gDNAase) (TIANGEN), according to the manufacturer’s instructions. Then, cDNAs were amplified with a fluorescence ratio PCR instrument (Roche 96) in a reaction with a final volume of 20 μl containing cDNAs (1 μl), YBR FAST qPCR Master Mix (10 μl), and primers (0.6 μl). A 5 min precycle at 95 °C was followed by 40 cycles of 15 s at 95 °C, 20 s at 60 °C and 15 s at 72 °C. The primer pairs for cDNA amplification were: 5’-CCG AGG CCC TAG CTT TCT TC-3′(forward) and 5′-GAG GAT CTT GAG GCT GGA CC-3′(reverse) for human CXCR4 (128 bp); 5’-GGC TTG CCT GGA CTT CTG TA-3′ (forward) and 5’-GTC AGC ACT ATG CCT CCC AA (reverse) for human CXCR7 (168 bp); 5′-CAT CAC CAT CTT CCA GGA GCG A-3′ (forward) and 5′-GTC TTC TGG GTG GCA G TG ATG G -3′ (reverse) for GAPDH(341 bp). Human first-trimester villi were used as a positive control. RNase-Free ddH_2_O was used as negative control. The housekeeping gene GAPDH was served as internal control. The experiments were performed on six samples by Beijing Yuan Quan Yi Ke Bio-Tech Co., Ltd.

### Western blotting

Proteins were extracted with pre-cooled RIPA buffer. After protein concentrations were measured using a BCA protein assay kit (SaiNuoBo-BIO, Beijing, China), cell lysates containing 10 μg of total protein were mixed with loading buffer, boiled at 95 °C for 5 min and loaded onto polyacrylamide-bisacrylamide gels (5% stacking gel and 12% separating gel). After the sodium dodecyl sulfate-polyacrylamide gel electrophoresis (SDS-PAGE), proteins were transferred to nitrocellulose membranes (Millipore Corporation, USA). Membranes were blocked with 5% fatty acid-free milk-TBST solution (Tris-buffered saline containing 0.1% Tween 20, pH 7.4) for 30 min at room temperature, and then incubated with rabbit anti-human CXCR4 (Abcam, Cambridge, UK, ab124824, diluted 1:500 in 5% fatty acid-free milk-TBST) or CXCR7 (diluted 1:500 in 5% fatty acid-free milk-TBST) antibodies overnight at 4 °C. After 5 washes with TBST solution, membranes were incubated with a peroxidase-conjugated goat anti-rabbit IgG secondary antibody (Beijing TDY BioTech Co. Ltd., diluted 1:10000 in fatty acid-free milk 5% in TBST) for 40 min at room temperature. β-actin antibody (Beijing TDY BioTech Co. Ltd., diluted 1:5000 in fatty acid-free milk 5% in TBST) was used as control. Proteins were revealed by Immobilon Western chemiluminescent HRP substrate (Millipore, MA, USA). Integrated optical densities of protein bands were measured using the Image Master VDS software. Experiments were performed on six samples at SINOBLE Biotechnology Center.

### Flow cytometry

Freshly isolated DECs were collected for the FCM analysis as previously described [13]. Briefly, cells were washed with phosphate-buffered saline (PBS) three times. After blocking with 10% FBS, cells were mixed with mouse anti-human CXCR4-PE-CY5 (5 μL/100 μL, catalogue number 15–9999-42, eBioscience, CA, USA,) or CXCR7-PE monoclonal antibody (5 μL/100 μL; clone 8F11-M16, Biolegend, San Diego, CA, USA). The corresponding mouse immunoglobulin (Ig) G2a-PE-CY5 or (Ig) G2a-PE isotype control was used as a negative control. After 30 min incubation in the dark at room temperature, the number of CXCR4, CXCR7 positive cells were measured using FACS flow cytometry system (BD FACS Canto II USA). The experiments were run duplicate in five different samples.

### Cell viability assay

The CCK-8 kit (Cell Counting Kit, DOJINDO Laboratories, Japan) assay was applied to evaluate the effects of CXCL12 on cell viability, as described previously [12]. Briefly, freshly isolated TCs were seeded in 6-well plates pre-coated with ECM at a density of 1 × 10^6^ cell/ml per well and cultured continuously for 48 h. Supernatants, namely TC-CM, were collected, centrifuged at 2000 g, and then stored at − 70 °C. The isolated DECs were re-suspended in DMEM supplemented with 15% FBS and seeded in 96-well flat-bottom microplates at a density of 2 × 10^4^ cells/well. After reaching 70–80% confluence, cells were starved with DMEM containing 1% FBS for 12 h before treatment. The medium was removed once again, and cells were treated with rh CXCL12 (100 ng/ml, catalogue number 300-28A-100, PeproTech, USA,) or TC-CM for 48 h. Before this stimulation, some of the wells with cells were pre-incubated with CXCL12 (25 μg/ml, catalogue number AF-351-NA, R&D Systems, Abingdon, UK), CXCR4 (20 μg/ml, catalogue number MAB172, R&D Systems, Abingdon, UK) or CXCR7 (10 μg/ml, catalogue number K0223–3, MBL international, Japan) neutralizing antibodies. Then, CCK-8 reagent (1:10) was added to the wells and incubated at 37 °C for 1 h. Absorbance was measured at a wavelength of 450 nm using an automatic microplate reader. Samples were run in triplicate and experiments were performed using three different samples.

### Migration and invasion assays

The migration and invasion of DECs were evaluated objectively with transwell plates, according to a previous study [12, 13]. For the migration assay, isolated DECs were directly seeded on the upper chamber (8 mm pore size, 6.5 mm diameter; Corning, NY, USA). For the invasion assay, isolated DECs were plated on the cell inserts pre-coated with 20 μl ECM (BD Matrigel™, Basement Membrane Matrix, USA) and two sets of experiments were performed as described below.

First, freshly isolated DECs (2 × 10^5^ cells in 200 μl of DMEM) were treated with rh CXCL12 (100 ng/ml). Before this stimulation, some of the wells with cells were pre-incubated with CXCR4 (20 μg/ml, catalogue number MAB172, R&D Systems, Abingdon, UK), CXCL12 (25 μg/ml, catalogue number AF-351-NA, R&D Systems, Abingdon, UK) or CXCR7 (10 μg/ml, catalogue number K0223–3, MBL international, Japan) neutralizing antibodies. The lower chamber was filled with 800 μl of DMEM supplemented with 10–15% FBS. Cells were then incubated at 37 °C for 48 h. Cultures of DECs alone were used as control.

Second, the TC and DEC co-culture invasion model was established to observe whether the invasiveness of DECs was regulated by TC-derived signals. Briefly, isolated TCs (2 × 10^5^ cells in 800 μl of DMEM supplemented with 15% FBS) were pre-seeded in the lower chambers and cultured for 24 h at 37 °C. Then, freshly isolated DECs (2 × 10^5^ in 200 μl DMEM) that had been pre-blocked with CXCR4 (20 μg/ml), CXCL12 (25 μg/ml) or CXCR7 (10 μg/ml) neutralizing antibodies were plated in the upper chamber. Cells were then incubated at 37 °C for an additional 48 h. Cultures of DECs alone were used as control.

The inserts were removed, washed with PBS and the non-invading cells and ECM were removed from the upper surface of the filter by wiping it with a cotton bud. The inserts were then fixed with 4% paraformaldehyde for 24 h and treated with 0.1% Triton X-100 for 40 min at room temperature. After blocking with goat serum for 2 h at room temperature, the inserts were incubated with primary anti-human keratin 7 antibody (1: 50, catalogue number 15539–1-AP, Protein Tech, Chicago, IL, USA,) overnight at 4 °C. After three washes with PBS, the inserts were incubated with biotinylated secondary antibody for 30 min at 37 °C and then incubated with DAPI (Santa Cruz Biotechnology) for 5 min at 37 °C. Images were captured with an Olympus fluorescence microscope (Olympus, Tokyo, Japan). The number of cells that had migrated to the lower surface was counted at a magnification of 200. Results were assessed by two independent researchers to eliminate inter-individual variability, and the invasive index was calculated as the proportion of the migrated cells of the experiment group relative to its own control. Each group was performed in duplicate and the experiment was repeated by 3 samples.

### Statistical analysis

Statistical comparisons of the data from the various groups were performed using one-way ANOVA followed by Dunnett’s post hoc t-test, when appropriate. Data are expressed as means ± SEM. Differences between groups were considered statistically significant at *P* < 0.05.

## Results

### Expression of CXCR4/CXCR7 in human first-trimester DECs

**Fig. 1:**
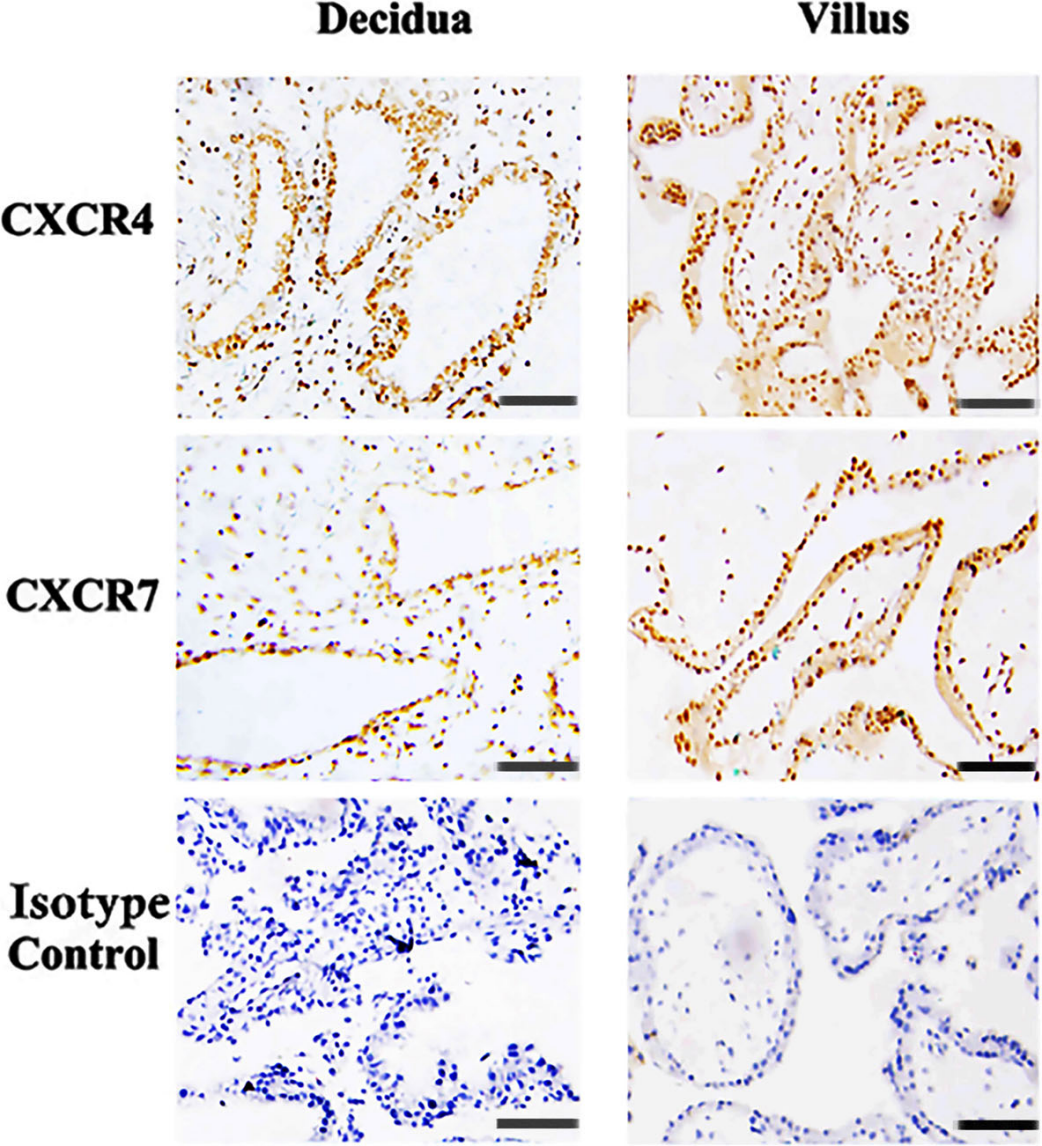
CXCR4 and CXCR7 were co-expressed in human first-trimester decidual epithelial cells in vivo. Immunohistochemistry expression of CXCR4 and CXCR7 molecules in first-trimester human decidual epithelial cells (DECs). Specific brown-colored staining for CXCR4 and CXCR7 were observed in the membrane, cytoplasm and nucleus of the primary-cultured DECs (*n* = 10). No staining was observed in cells stained with murine isotype control antibodies. Human first-trimester villi were used as positive controls. Murine isotype represents a negative control. Experiments were performed using ten independent endometrial samples. —200 μm

**Fig. 2:**
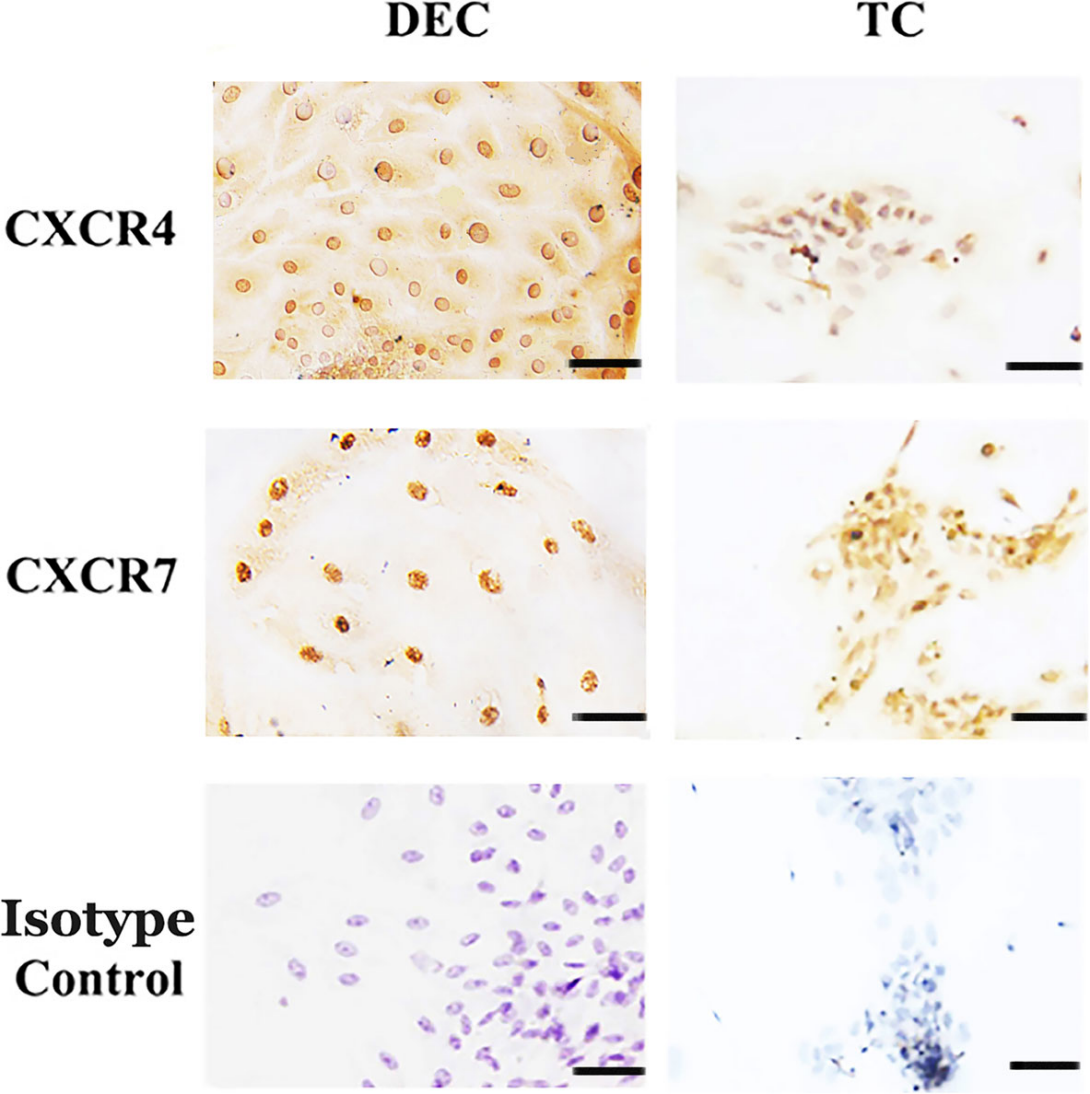
CXCR4 and CXCR7 were co-expressed in primary human first-trimester decidual epithelial cell cultures in vitro. Immunocytochemistry detection of CXCR4 and CXCR7 expression in first-trimester human primary cultured decidual epithelial cells (DECs). Specific staining against CXCR4 or CXCR7 was observed in the membrane, cytoplasm and nucleus of the DECs (*n* = 5). No staining was observed in cells stained with murine isotype control antibodies. Human first-trimester cultured cytotrophoblasts were used as positive controls. Magnification: × 200. Experiments were performed using five independent endometrial samples. —50 μm

The immunochemical staining shown in Figs 1 and 2 revealed a brown-colored staining specific for CXCR4 or CXCR7 in the cytomembrane, cytoplasm and nucleus of human first-trimester DECs in vivo and in vitro.

**Fig. 3:**
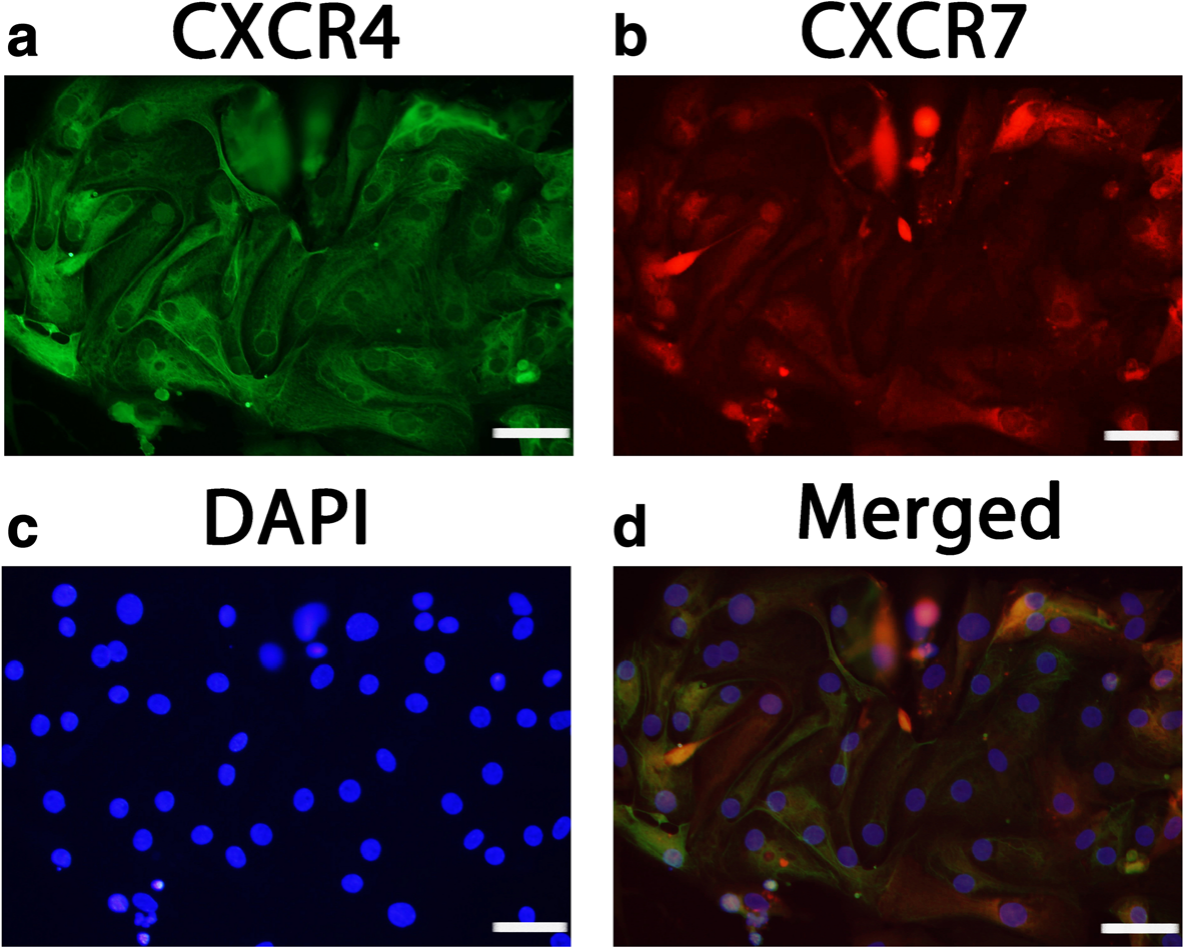
CXCR4 and CXCR7 were co-expressed in primary human first-trimester DECs cultures in vitro. Immunofluorescence detection of both CXCR4 and CXCR7 protein were expressed in the membrane, cytoplasm and nucleus of DECs. **a** Expression of CXCR4 (green fluorescence) in DECs; **b** Expression of CXCR7 (red fluorescence) in DECs; **c** DAPI staining in DECs; **d** The merged picture. Magnification: × 400. Experiments were performed using three independent endometrial samples. —50 μm

The immunofluorescence results in Fig. 3 showed that CXCR4 and CXCR7 protein were co-expressed in human in vitro cultured DECs. Both the red fluorescence (CXCR7) and green fluorescence (CXCR4) was clearly recognized in the same area of the cells, including the cytomembrane, cytoplasm and nucleus.

**Fig. 4:**
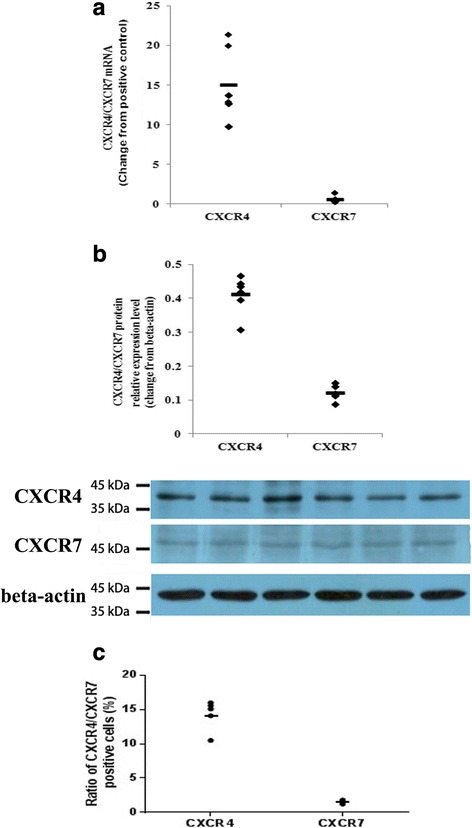
Expression levels of CXCR4 and CXCR7 in primary human first-trimester decidual epithelial cell cultures. Expression of the CXCR4 and CXCR7 mRNAs and proteins was detected in primary human first-trimester decidual epithelial cell (DEC) cultures. **a** The expression of the CXCR4 and CXCR7 genes in DECs was examined by real-time PCR. The level of the CXCR4 or CXCR7 gene was equal to the ratio of the absorbance of the target gene to the control. The transverse line represents the average CXCR4 and CXCR7 levels in DECs. (*n* = 6). **b** The expression of CXCR4 and CXCR7 proteins in DECs was detected by western blotting. (*n* = 6). **c** Flow cytometry results of the expression of CXCR4 and CXCR7 on the membranes human first-trimester DECs (*n* = 5). The transverse line represents mean percentage of CXCR4 or CXCR7 positive cells

As shown in the real-time PCR results presented in Fig. 4a, both the CXCR4 and CXCR7 genes were transcribed in human first-trimester DECs in vitro, and the mean levels of the CXCR4 and CXCR7 mRNAs in DECs were 15.0877 and 0.6158, respectively, changed from positive control.

The expression of the CXCR4 and CXCR7 proteins in DECs was detected by western blotting. As shown in Fig. 4b, the CXCR4 and CXCR7 proteins were co-expressed in human first-trimester DECs. The relative intensities of the CXCR4 and CXCR7 proteins were 0.4135 ± 0.1538 and 0.1222 ± 0.0219, respectively.

We used flow cytometry to detect the expression of CXCR4 and CXCR7 on the membranes of human first-trimester DECs from 5 independent samples. CXCR4 and CXCR7 proteins were detected in all of the analyzed samples. The the percentage of CXCR4-positive cells varied from 10.5% to 16.0%, with an average of 14.26 ± 1.00%, while the percentage of CXCR7-positive cells ranging between 1.2 and 1.8%, with an average of 1.50 ± 0.11% (Fig. 4c).

### The soluble CXCL12 content in TC-CM

**Fig. 5:**
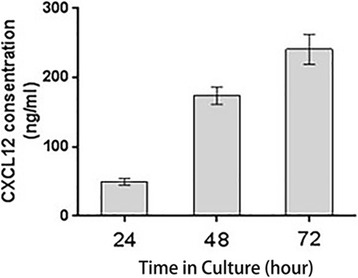
Primary human first-trimester trophoblast cultures spontaneously secreted CXCL12 in vitro. Spontaneous CXCL12 secretion in supernatants of in vitro cultured TCs measured at 24, 48 and 72 h in culture.(*n* = 3)

The primary cultured human first-trimester TCs secreted CXCL12 spontaneously in vitro, as shown in ELISA assays (Fig. 5). The concentration of CXCL12 increased with time in culture: it was 49.57 ± 2.42 ng/ml, 173.80 ± 6.22 ng/ml and 240.77 ± 10.83 ng/ml at 24, 48 and 72 h in culture.

### The CXCL12/CXCR4/CXCR7 axis had no effect on the viability of human first-trimester DECs in vitro

**Fig. 6:**
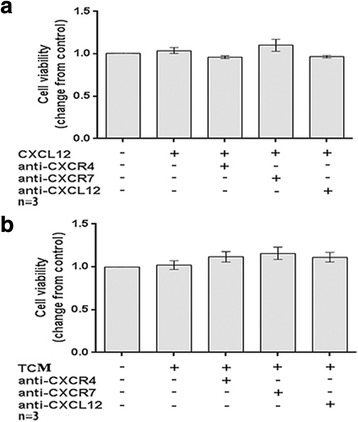
The CXCL12/CXCR4/CXCR7 axis had no effect on the viability of human first-trimester DECs in vitro*.* The CCK-8 assay was used to explore the effects of the CXCL12/CXCR4/CXCR7 axis on decidual epithelial cell (DEC) viability. The viability index was changed compared to the corresponding control. **a** The rhCXCL12 treatment had no detectable effect on the viability of human first-trimester DECs in vitro. **b** Effects of TCM from trophoblasts on DEC viability in vitro. CXCL12: treated with 100 ng/ml recombinant human CXCL12; anti-CXCR4, treated with 20 μg/ml CXCR4 neutralizing antibody; anti-CXCR7, treated with 10 μg/ml CXCR7 neutralizing antibody; anti-CXCL12, treated with 25 μg/ml CXCL12 neutralizing antibody; TCM: trophoblast-conditioned medium. Data are presented as average ± SEM. (*n* = 3)

As shown in Fig. 6, neither CXCL12 (1.04 ± 0.04, *P* > 0.05) nor TC-CM (1.02 ± 0.05, *P* > 0.05) increased the viability of DECs in vitro. Likewise blocking the CXCL12, CXCR4 or CXCR7 also failed to affect DEC viability (*P* > 0.05).

### TC-derived CXCL12 promoted the migration and invasion of human first-trimester DECs by binding to CXCR4, rather than CXCR7

**Fig. 7:**
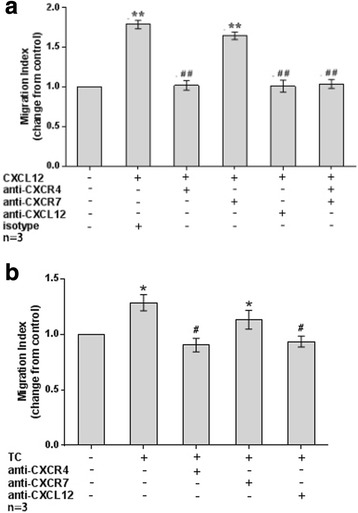
TC-derived CXCL12 induced DEC migration by binding to CXCR4 rather than CXCR7. **a** The addition of CXCL12 significantly increased DECs migration in vitro*,* while neutralizing antibodies to CXCR4 or CXCL12 effectively inhibited the CXCL12-stimulated DEC invasiveness. The CXCR7 antibody failed to block the CXCL12-induced increase in DEC migration. (*n* = 3). **b** Co-culture with TCs significantly increased the DECs migration in vitro*,* and a neutralizing antibody against CXCR4 or CXCL12 effectively inhibited the CXCL12-stimulated DEC migration. The CXCR7 antibody failed to block the TC co-culture-induced increase in DEC migration. * *P* < 0.05, ** *P* < 0.01, compared to the control; # *P* < 0.05, ## *P* < 0.01, compared to the CXCL12-treated group or TC co-culture group. Data are presented as average ± SEM. (*n* = 3)

In the migration assay (Fig. 7), both exogenous CXCL12 (1.79 ± 0.05, *P* < 0.01) and co-culture with TC (1.53 ± 0.08, *P* < 0.05) significantly increased the migration of human first-trimester DECs in vitro compared to the corresponding control. CXCL12 (1.79 ± 0.05 vs 1.02 ± 0.06 and 1.01 ± 0.07, respectively for control, *P* < 0.01) or TC co-culture-induced migration (1.53 ± 0.08 vs 1.14 ± 0.05 and 1.11 ± 0.02, respectively for control, *P* < 0.05) was decreased after treatment with neutralizing antibodies against CXCR4 or CXCL12. However, the blockade of CXCR7 failed to inhibit the CXCL12 (1.79 ± 0.05 vs 1.64 ± 0.05, *P* > 0.05) or TC co-culture-induced (1.53 ± 0.08 vs 1.46 ± 0.07, *P* > 0.05) increase in migration.

**Fig. 8:**
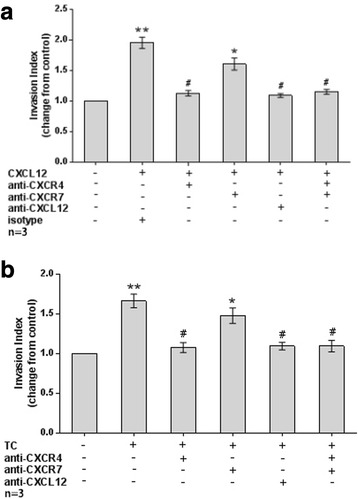
TC-derived CXCL12 increased DEC invasion by binding to CXCR4 rather than CXCR7. **a** The addition of CXCL12 significantly increased the DECs invasion in vitro*,* while neutralizing antibody against CXCR4 or CXCL12 effectively inhibited the CXCL12-stimulated DEC invasion. The CXCR7 antibody failed to block the CXCL12-induced increase in DEC invasion. (*n* = 3). **b** Co-culture with TCs significantly increased DEC invasion in vitro*,* and a neutralizing antibody against CXCR4 or CXCL12 effectively inhibited the TC co-culture-stimulated DEC invasion. The CXCR7 antibody failed to block the TC co-culture-induced increase in DEC invasion. * *P* < 0.05, ** *P* < 0.01, compared to the control; # *P* < 0.01, compared to the CXCL12-treated group or TC co-culture group. Data are presented as average ± SEM. (*n* = 3)

The results of the invasion assay (Fig. 8) were similar to the migration assay. The number of invaded DECs was significantly increased after treatment with exogenous CXCL12 (1.96 ± 0.09 vs 1.00, *P* < 0.01) or co-culture with TC (1.67 ± 0.09 vs 1.00, *P* < 0.01) compared with the corresponding controls. Addition of antibodies against CXCR4 or CXCL12 effectively blocked the CXCL12- stimulated invasion of DECs (1.96 ± 0.09 vs 1.13 ± 0.04 and 1.09 ± 0.03, *P* < 0.01) as did the TC co-culture (1.67 ± 0.09 vs 1.08 ± 0.06 and 1.10 ± 0.05, *P* < 0.01). Treatment with the CXCR7 neutralizing antibody alone was unable to block the increased invasion induced by exogenous CXCL12 (1.96 ± 0.09 vs 1.61 ± 0.10, *P* > 0.05) or TC co-culture (1.67 ± 0.09 vs ± 1.48 ± 0.10, *P* > 0.05). Although the invasive index of the CXCR4 + CXCR7 combination group was significantly lower than the CXCL12 group (1.96 ± 0.09 vs 1.15 ± 0.04, *P* < 0.01) or TC co-culture group (1.67 ± 0.09 vs 1.10 ± 0.07, *P* < 0.01), no significant differences in invasion were observed between the CXCR4 alone and CXCR4 + CXCR7 combination groups [1.13 ± 0.04 vs 1.15 ± 0.04, *P* > 0.05 (CXCL12 treatment); 1.08 ± 0.06 vs 1.10 ± 0.07, *P* > 0.05 (TC co-culture)].

## Discussion

In the present study, we confirmed the co-expression of CXCR4 and CXCR7 in human DECs in situ and in in vitro cultures at both the mRNA and protein levels. Fetal-derived TCs are the main sources of CXCL12 [12, 13, 19, 20]. Thus, it is reasonable to infer that at maternal-fetal interface, the co-expression of two receptors for a common fetal-derived chemokine in DECs might be an important mechanism by which the maternal endometrium can better coordinate with the embryo/fetus during implantation and early pregnancy via the timely “sensing” of some fetal-derived signals by maternal-derived cells.

The in vitro viability of DECs was not affected by treatment with CXCL12 or TC-CM, supporting previous findings from our team showing that the CXCL12/CXCR4 axis had no effect on the viability or PCNA expression of DSCs in vitro [12, 13]. However, in contrast to DECs and DSCs, TCs increase their own viability in vitro through CXCL12/CXCR4 binding [21]. CXCL12/CXCR4/CXCR7 signaling has also been shown to mediate the growth of several types of tumors [22–24]. Thus, a question is raised: why is the CXCL12/CXCR4/CXCR7 axis not required for the viability of DECs or DSCs, when they are stably co-expression in these two cell types? We postulate that this signaling pathway might exert different effects on different types of cells. During implantation and placentation, other biological functions of the maternal-derived cells rather than proliferation and viability might be more important for the crosstalk with the fetal-derived TCs. Of course, this assumption requires further study.

A unique strength of this study is the investigation into the modulatory effects of TCs on the motility of DECs via the CXCL12/CXCR4/CXCR7 axis. An enormous amount of research effort has examined the invasion of TCs but has neglected the motile characteristics of other type of cells at the maternal-fetal interface [25, 26]. Our knowledge of the characteristics of DEC, particularly whether DECs have the ability to migrate or invade during early pregnancy, has remained an open question until now. In the present study, DECs from early pregnancy are capable of spontaneously migrating and invading in vitro, and these abilities are further increased after stimulation with a TC-derived signal (CXCL12) and CXCR4 binding. According to our previous study, human first-trimester DSCs also migrate after activation of the CXCL12/CXCR4 signaling pathway [12, 13]. Obviously, as the main maternal-derived cells at the maternal-fetal interface, both DECs and DSCs initially display motile potential after “sensing” the signal released by TCs (CXCL12) via the co-expression of the same receptor, CXCR4.

Recently, several researchers have begun to focus on the regulation of the decidua and control of TC invasion [4, 25, 27]. In animal studies, focal adhesion proteins undergo dynamic changes in their distributions in uterine luminal epithelial cells that disassembled from the site of focal adhesions at the time of implantation, facilitating the removal of uterine luminal epithelial cells for embryo invasion [28, 29]. Together with these studies, our results have caused us to re-examine some previous views on the role of the endometrium and have provided a new perspective of maternal-fetal interactions during implantation: the decidual cells, to some degree, can make way for the invading trophoblast cells.

In our opinion, the endometrium might be dynamically motile and make positive adjustments rather than merely passively waiting for the embryo to better accept the arrival of the embryo. The ability of DECs to migrate is important for obtaining a better understanding of endometrial receptivity, implantation and clinical treatments for recurrent implantation failure.

As a newly identified second receptor for CXCL12, the biological function of CXCR7 is much more complicated and controversial than CXCR4 [19, 23, 24, 30–33]. Some researchers have postulated that CXCR7 acts as a decoy receptor because of its inability to induce calcium mobilization, cell migration or integrin activation upon binding to CXCL12, [20, 23, 30] whereas others argue against this hypothesis due to the observed roles of CXCR7 in signaling activity and migration, particularly in tumor growth and metastasis [23, 31–33]. In the present work, the addition of CXCR7 neutralizing antibody failed to block the TC-induced increase in DEC invasion. However, treatment with CXCR4 or CXCL12 neutralizing antibody significantly decreased the invasive index, but no differences in the blocking effects were observed among the CXCR4 alone, CXCL12 alone and CXCR4 + CXCR7 combination groups. Thus, despite the co-expression of CXCR4 and CXCR7 in DECs, the effects of TC-derived CXCL12 on promoting DEC migration and invasion are mainly mediated by CXCR4, rather than CXCR7. The lack of CXCR7 function in CXCL12/CXCR4-mediated DEC invasion suggests that CXCR7 has a decoy function at the maternal-fetal interface. Thus, the cell type may determine whether CXCR7 activates signaling or sequesters CXCL12 upon binding.

Notably, unlike CXCR4, CXCR7 is also a receptor for CXCL11 (IFN-inducible T cell alpha chemoattractant (I-TAC), [20] and CXCL11 has been confirmed to be expressed in the uterus during the peri-implantation period [33, 34]. Moreover, CXCR3, another receptor for CXCL11, is also expressed at the maternal-fetal interface, and CXCL11/CXCR3 signaling mediates the migration of TCs and T cells [33, 34]. Therefore, we cannot exclude the possibility that CXCL12/CXCR7-mediated responses are potentially modulated by CXCL11/CXCR3 signaling. The complexity of the chemokine network at the maternal-fetal interface might be beyond our imagination, and more research is required to elucidate the interaction between the fetus and endometrium during implantation and early pregnancy. Additionally, it is very difficult for us to obtain sufficient number of abortion tissues and the sample size limits the data analysis and the soundness of the present results. Thus, there is still a long way to unveil the role of chemokine and chemokine receptor at the mateo-fetal interface.

## Conclusions

As shown in the present study, CXCR4 and CXCR7 are co-expressed in human first-trimester DECs and TC-derived CXCL12 promotes the migration and invasion of DECs by binding to CXCR4, rather than CXCR7. The discovery that TC-released signals are capable of modulating the migratory and invasive behaviors of DECs contributes to a better understanding of the crosstalk in different type of cells at the maternal-fetal interface during implantation and early pregnancy, and provides new clues for the clinical treatment for recurrent implantation failure and early miscarriage.

## Abbreviations

CXCL12: C-X-C motif chemokine ligand 12
CXCR4: C-X-C chemokine receptor type 4
CXCR7: C-X-C chemokine receptor type 7
DAB: 3, 30-diaminobenzidine tetrahydrochloride
DEC: Decidual epithelial cell
DMEM: Dulbecco’s Modified Eagle’s Medium
DSC: Decidual stroma cells
ELISA: Enzyme-linked immunosorbent assay
FBS: Fetal bovine serum
ICC: Immunocytochemistry
IHC: Immunohistochemistry
PBS: Phosphate-buffered saline
PCR: Polymerase chain reaction
rhCXCL12: Recombinant human CXCL12
TBST: Tris-buffered saline with Tween 20
TC: Trophoblast cell
TC-CM: TC-conditioned medium
